# Hydroalcoholic Extract from Inflorescences of* Achyrocline satureioides *(Compositae) Ameliorates Dextran Sulphate Sodium-Induced Colitis in Mice by Attenuation in the Production of Inflammatory Cytokines and Oxidative Mediators

**DOI:** 10.1155/2016/3475356

**Published:** 2016-10-25

**Authors:** Luisa Mota da Silva, Jaime Antonio Machado Farias, Thaise Boeing, Lincon Bordignon Somensi, Ana Paula Beber, Benhur Judah Cury, José Roberto Santin, Sérgio Faloni de Andrade

**Affiliations:** Programa de Pós-Graduação em Ciências Farmacêuticas (PPGCF), Núcleo de Investigacões Químico-Farmacêuticas (NIQFAR), Universidade do Vale do Itajaí (UNIVALI), Rua Uruguai, 458, Centro, 88302-202 Itajaí, SC, Brazil

## Abstract

*Achyrocline satureioides* is a South American herb used to treat inflammatory and gastrointestinal diseases. This study evaluated intestinal anti-inflammatory effects of the hydroalcoholic extract of inflorescences of* satureioides *(HEAS) in dextran sulfate sodium (DSS) induced colitis in mice. Mice were orally treated with vehicle, 5-aminosalicylic acid (100 mg/kg), or HEAS (1–100 mg/kg). Clinical signs of colitis and colonic histopathological parameters were evaluated, along with the determination of levels of reduced glutathione and lipid hydroperoxide (LOOH), the superoxide dismutase (SOD), and myeloperoxidase (MPO) activity in colon. The colonic content of cytokines (TNF, IL-4, IL-6, and IL-10) was measured. Additionally, the effects of the extract on nitric oxide (NO) release by lipopolysaccharide (LPS) stimulated macrophages and diphenylpicrylhydrazyl levels were determined. Mucin levels and SOD activity, as well as the LOOH, MPO, TNF, and IL-6 accumulation in colon tissues, were normalized by the HEAS administration. In addition, the extract elicited an increase in IL-4 and IL-10 levels in colon. NO release by macrophages was inhibited by HEAS and its scavenger activity was confirmed. Together these results suggest that preparations obtained from inflorescences from* A. satureioides* could be used in treatment for IBD. Besides, this work corroborates the popular use of* A. satureioides* in inflammatory disorders.

## 1. Introduction

Ulcerative colitis (UC) is chronic inflammatory bowel disease (IBD) characterized by diffuse inflammation of the rectal and colonic mucosa [1]. The incidence and prevalence of this chronic condition have increased worldwide but diverge widely among geographic regions [2]. The precise etiology of ulcerative colitis is unknown, but it is thought that mucosal inflammation is the result of an abnormal colonic immune response and interactions between genetics, colonic gut flora, and environmental factors [3]. Patients with inflammation in the intestinal mucosa complain of symptoms such as bloody diarrhea, abdominal pain, urgency, and tenesmus [4].

The conventional treatment of UC includes the use of anti-inflammatory drugs (aminosalicylates or corticosteroids), immunosuppressant, antibiotics, and biologic agents [5]. This therapy is carried out by a step-up strategy in accordance with severity of symptoms aiming to induce and maintain the disease remission and improve the quality of life [4]. In addition, the therapy also decreases the risk of complications, such as developing colorectal cancer [6]. However, drug refractoriness, intolerance, major adverse events, and poor treatment responses are related to the current therapy of UC [7]. In this scenario, treatment modalities that effectively attenuate the bowel inflammation due to UC with fewer adverse effects are need. The use of herbal therapy in inflammatory bowel diseases (IBD) is increasing worldwide, and it is clear that alternative or complementary medicine is an interesting source for the discovery of new, more effective, and safe drug.

*Achyrocline satureioides* (Lam.) DC (Compositae) is a medium-sized South American indigenous herb distributed in Europe and Africa popularly known as “Marcela” or “Macela” [8]. Infusions of the inflorescences from this species are used in Brazilian folk medicine to treat central nervous system diseases, respiratory diseases, inflammatory disorders, and gastrointestinal system diseases [9]. More particularly, there are several reports indicating that tea prepared mainly by communities of South Brazil, from the inflorescences of* A. satureioides*, leads to the relief of the symptoms of gastric ulcers [10, 11] and inflammatory diseases of the gastrointestinal tract, such as Crohn's disease [11, 12]. In line with its traditional use, the anti-inflammatory activity of extracts from* A. satureioides *has been confirmed in different studies [11, 13, 14]. Santin et al. [15] previously confirmed the antiulcer gastric potential of this plant and its antispasmodic effect in gastrointestinal smooth muscle preparation has also been described [13]. Moreover, no signs of toxicity were detected after the administration of the hydroalcoholic extract from inflorescences of* A. satureioides *at a dose of 2000 mg/kg in female rats [15]. However, despite these findings and popular usage to treat IBD, no reports are available about the intestinal anti-inflammatory effects* A. satureioides *preparations. Therefore, the present study has been designed to examine the intestinal anti-inflammatory effects of the hydroalcoholic extract of inflorescences of* A. satureioides *(HEAS), which has already been chemically characterized being the major components identified as quercetin and luteolin [14], in dextran sulfate sodium (DSS) induced colitis in mice.

## 2. Material and Methods

### 2.1. Chemicals

The following substances were used: 2,2-diphenyl-1-picrylhydrazyl, 5,5′-dithiobis (2-nitrobenzoic acid), bovine serum albumin, glutathione, butylated hydroxytoluene, Griess reagent, MTT, pyrogallol, xylenol orange (all from Sigma, St. Louis, USA), absolute ethanol, acetic acid, ascorbic acid, ferrous ammonium sulfate, hydrochloric acid, formaldehyde, hydrogen peroxide, methanol, sodium acetate, trichloroacetic acid (Vetec, Rio de Janeiro, RJ, Brazil), sulfuric acid, dimethyl sulfoxide and N,N-dimethylformamide (DMSO, Synth, Diadema, SP, Brazil), Dulbecco's Modified Eagle Medium (DMEM, Vitrocell, Campinas, SP, Brazil), fetal bovine serum (FBS, Gibco), and dextran sulfate sodium (Alfa Aesar, Heysham, Lancashire, UK).

### 2.2. Plant Material, Preparation of the Extract, and Phytochemical Identification

Inflorescences of* A. satureioides *were collected in Fraiburgo, Santa Catarina, Brazil. A voucher specimen is deposited at the herbarium of the Universidade Estadual de Maringá (UEM) under the number HUEM-23568. Professor Oscar Iza authenticated the sample.

The extraction procedures had already been published by Barioni et al. [14]. Air-dried inflorescences (2 kg) were cut into small pieces and macerated with 70% (v/v) aqueous ethanol (10 L) at room temperature for 7 days. The hydroalcoholic extract of inflorescences of* A. satureioides *(HEAS) was obtained by filtration of the macerated material and solvent evaporation under reduced pressure, yielding 192.6 g (9.6%) of hydroalcoholic extract.

The phytochemical composition of HEAS was also previously described by Barioni et al. [14], and the flavonoids luteolin and quercetin were identified as the major constituents.

Aiming to establish standardization, the total phenolic and flavonoid content were quantified in HEAS. The total phenolic content was verified by Folin-Ciocalteu reagent according to the method described by Arnous et al. [16]: HEAS (50, 100, 150, and 200 *μ*g/mL) was mixed with 0.5 mL of distilled water and 2.5 mL of Folin-Ciocalteu reagent (1 : 10 dilution) and 2.0 mL of sodium carbonate (7.5% w/v) were added in the tubes. Further, the tubes were incubated at 45°C for 15 min. The absorbance was determined at 760 nm in spectrophotometer. The total polyphenol concentration was calculated from a calibration curve, using tannic acid as a standard. Results were expressed as tannic acid equivalents (TAE) in *μ*g.

The flavonoid content was estimated by the AlCl_3_ method [17]: 1 mL of methanolic extract solution (50–200 *μ*g/mL) was added to equal volume of 2% methanolic AlCl_3_, 6H_2_O. The absorbance was measured 10 min later at 430 nm. The results were expressed in mg quercetin/100 g dry extract by comparison with standard quercetin treated in the same conditions.

### 2.3. Animals

Swiss female mice (25–30 g) and female Wistar rats (180–200 g) were purchased from animal house of the Universidade do Vale do Itajai (UNIVALI), Itajaí, SC, Brazil. All mice were housed six per cage in the animal room at 22 ± 2°C under a 12 h light/12 h dark cycle and with access to food and water* ad libitum*. All experimental animal procedures were approved by the Institutional Ethics Committee of the UNIVALI under approval certificate number 035/15 and were carried out in accordance with the international standards and the ethical guidelines on animal welfare.

### 2.4. Dosage Fixation

The doses of HEAS used in this study were 1, 10, and 100 mg/kg administered by oral route. This range of doses was based on data previously published by Barioni et al. [14] and Santin et al. [15], which addressed the gastroprotective and the anti-inflammatory effects of HEAS in doses established in folk medicine.

### 2.5. Induction of Colitis

**Figure 1:**
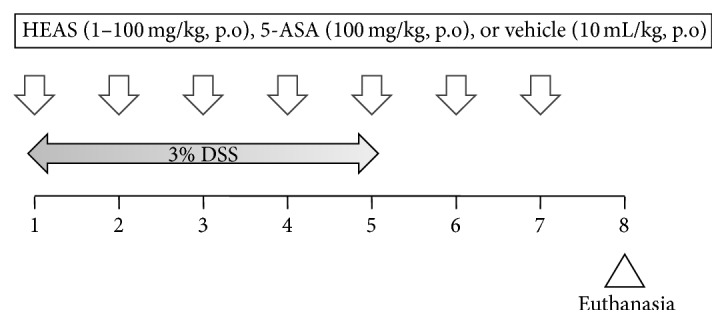
Experimental protocol. The animals were treated with vehicle or HEAS (100 mg/kg, po) daily for seven days. Simultaneously, the animals received 3% DSS from 1st to 5th day of treatment.

Mice were randomly divided into three groups (*n* = 10). Colonic inflammation was induced by addition of 3% (w/v) DSS (MW: 40,000) in drinking water* ad libitum* for 5 days, as described by Chassaing et al. [18]. Noncolitic group did not receive DSS in drinking water. Control-colitic group was treated once a day, for 7 days, with vehicle (water, 10 mL/kg). HEAS-treated colitic group received the extract (1, 10, and 100 mg/kg, po) once a day for 7 days. The positive control agent used in this experiment was 5-aminosalicylic acid (5-ASA), administered at a dose of 100 mg/kg (p.o) once a day for 7 days in colitic animals. The treatment with vehicle or extract started simultaneously with the DSS administration and the experimental protocol is showed in Figure 1. Animal body weight, the presence of rectal gross blood, and stool consistency were individually evaluated daily. Each parameter was assigned a score according to the criteria previously proposed [19, 20] and used to calculate on average daily the Disease Activity Index (DAI) (Table 1). The animals were euthanized at 8th day after the beginning of treatments, at which the colon was excised, weighed, and measured in length. In addition, representative specimens (0.5 cm length) containing all wall layers were taken from the proximal inflamed region and fixed in ALFAC solution (a mixture containing 80% alcohol, 15% formaldehyde, and 5% acetic acid) for the histological studies; equivalent colonic segments were also obtained from the noncolitic group. The remaining colonic tissue was subsequently sectioned in different longitudinal fragments to be used for inflammatory and oxidative parameters measurement. Furthermore, the liver and spleen from colitic and noncolitic mice were excised and weighed. Moreover, feces were collected for occult blood measurement by Mayer method and for intestinal bleeding score calculation [18].

### 2.6. Histological Studies

#### 2.6.1. Histopathological Analysis

 Tissue sections of colonic tissue were embedded in paraffin. Full thickness sections of 5 *μ*m were stained with hematoxylin and eosin (HE). After that, the slides were dehydrated, cleared, and mounted between slide and cover slip. The histological damage was evaluated by a pathologist observer, according to the criteria previously described by Utrilla et al. [19] and Camuesco et al. [20], with few modifications, and taking into account the presence of epithelial loss, cell infiltration, edema, and the condition of the crypts and Goblet cells. The colonic tissue was evaluated focusing on the previous features and a score ranging from 0 (healthy tissue) to 3 or 4 (severe damage), depending on the item, was assigned to each one (full details in Table 2). The sum gives the total score for each sample.

#### 2.6.2. Histochemical Analysis

For mucin content analysis, the histologic sections, obtained as described above, were oxidized in 0.5% periodic acid in water, at room temperature, for 5 min. The tissue was then washed in water, immersed in Schiff's reagent for 20 min, and rinsed in water for 5 min and then three times in 0.5% sodium meta-bisulphite before a final wash in water. After that, sections were stained with hematoxylin for 20 s, dehydrated, cleared, and mounted between slide and cover slip. Next, the slides were observed in optical microscope and photographed. In addition, the glycoproteins were quantified using the program Image J® and expressed as pixels/field.

### 2.7. Preparation of Subcellular Fractions of Tissues

Colon samples from the different experimental groups were homogenized with 200 mM potassium phosphate buffer (pH 6.5), and the homogenate was used to measure the reduced glutathione (GSH), lipid hydroperoxide (LOOH), and cytokines levels. After that, the homogenate was centrifuged at 4000 rpm for 20 min at 4°C, myeloperoxidase (MPO) activity was measured in the precipitate, and superoxide dismutase (SOD) activity was determined in supernatant.

### 2.8. Protein Assay

Protein levels were determined by the Bradford method (Bio-Rad, Hercules, CA, USA), using a standard curve of bovine serum albumin (0.1–0.0125 *μ*g/mL) as standard, according to instructions of the manufacturer.

### 2.9. Determination of Oxidative Parameters

#### 2.9.1. Determination of GSH Levels

GSH levels in colon were determined as previously described [21]. Briefly, aliquots of the homogenate prepared as described above were mixed with 12.5% trichloroacetic acid and centrifuged at 6000 rpm, 20 min. The absorbance of supernatant plus TRIS buffer (0.4 M, pH 8.9) and 5,5′-dithiobis 2-nitrobenzoic acid (DTNB, 0.01 M) was determined spectrophotometrically at 415 nm. The individual values were interpolated in a standard curve of GSH (1.25–10 *μ*g/mL) and expressed as mg/g of tissue. All procedures were performed at 4°C.

#### 2.9.2. Determination of LOOH Content

The levels of LOOH in colon were determined as previously described by the method of Ferrous Oxidation-Xylenol Orange (FOX2) [22]. In this assay, 100 *μ*L of supernatant plus 100 *μ*L of methanol P.A. was centrifuged at 13000 rpm for 5 minutes (4°C). FOX2 reagent (4 mM butylated hydroxytoluene (BHT), 250 mM FeSO_4_, 25 mM H_2_SO_4_, and xylenol orange at 100 mM) were added to supernatant and incubated, in dark, for 30 min at room temperature. The absorbance was determined spectrophotometrically at 560 nm, and the concentration of LOOH content was calculated using molar extinction coefficient (*E* = 43 mM^−1 ^cm^−1^) and expressed as mmol hydroperoxides/mg of tissue.

#### 2.9.3. Determination of SOD Activity

SOD activity was measured based on its ability to inhibit the pyrogallol autoxidation [23]. Briefly, aliquots of the supernatant were added to buffer solution (200 mM TrisHCl-EDTA, pH 8.5), and pyrogallol (1 mM) and then incubated for 20 min at room temperature. Further, HCl 1 N was added to stop the reaction and then the mixture was centrifuged for 4 min at 18700 ×g. The absorbance of the resulting supernatant was measured at 405 nm. The amount of SOD that inhibited the oxidation of pyrogallol by 50%, relative to the control, was defined as one unit of SOD activity. The SOD activity was expressed as U/mg of protein.

#### 2.9.4. *In Vitro* 2,2-Diphenyl-1-picrylhydrazyl (DPPH) Scavenging Activity

The DPPH assay has been widely used as a tool to estimate the free radical scavenging activity of antioxidants constituents in plant extracts. The reduction capacity of the DPPH radical was determined by the decrease in absorbance induced by antioxidants according to Blois [24] and Chen et al. [25], with modifications. Different concentrations of HEAS (1, 10, 100, and 1000 *μ*g/mL) were mixed with DPPH methanolic solution (10 *μ*g/mL). In this test, ascorbic acid (50 *μ*g/mL) was used as a positive control. The solutions were mixed and incubated for 5 min at room temperature and the absorbance was read at 517 nm. The individual values were interpolated to a standard curve of DPPH (0–60 *μ*M) and expressed as *μ*M de DPPH. All the experiments were performed in triplicate.

### 2.10. Determination of Inflammatory Parameters

#### 2.10.1. Measurement of* In Vivo* MPO Activity

Colonic and hepatic MPO activity were measured according to the method described by Bradley et al. [26] and modified by De Young et al. [27]. Briefly, precipitate of the homogenate prepared as described above was mixed in 80 mM potassium phosphate buffer (pH 5.4) containing 0.5% hexadecyltrimethylammonium bromide (HTAB) and centrifuged (12000 rpm for 20 min at 4°C). MPO activity was determined at 620 nm in the supernatant in presence of H_2_O_2_ and 3,3′,5,5′-tetramethylbenzidine (TMB). MPO activity was expressed as units of optic density (mO.D.)/mg of protein.

#### 2.10.2. Determinations of Cytokine Levels

Colonic homogenates were used to estimate the TNF-*α*, IL-6, IL-4, and IL-10 levels by enzyme-linked immunosorbent assay (ELISA), using mouse cytokine ELISA kits from BD Biosciences (Franklin Lakes, New Jersey, USA), according to the manufacturer's instructions. The absorbance was measured at 450 and 550 nm and the results were expressed as pg/mL.

#### 2.10.3. Nitric Oxide Determination in Isolated Rat Peritoneal Macrophage Stimulated with LPS

Untreated Wistar rats were given a volume of 5 mL intraperitoneal injection of 3% thioglycollate in PBS. Four days later, mouse peritoneal macrophages were collected through peritoneal lavage with PBS, pelleted, and washed in PBS. Cells were plated at a density of 1 × 10^6^ cells per mL with Dulbecco's Modified Eagle Medium (DMEM) supplemented with 10% fetal bovine serum, penicillin (100 U/mL), and streptomycin (100 U/mL). Cells were given 2 h to adhere, after which the medium was changed, and adherent cells were treated with HEAS (1, 10, and 100 *μ*g/mL) and stimulated with LPS (1 *μ*g/mL) for 24 h without removing the extract from the culture medium. The supernatant was used for nitric oxide measurement.

Nitric oxide levels were assessed by means of nitrite quantification as described by Grisham et al. [28]. Briefly, 100 *μ*L of culture medium was incubated for 15 min with Griess reagent. Absorbance was read at 540 nm.

### 2.11. Cell Viability Assay

Cell viability was determined using the MTT reduction assay. Peritoneal macrophages were seeded in a 96-well plate at density of 10^5^ cells/well and treated with different concentrations of HEAS (1, 10, and 100 *μ*g/mL) for 24 h. After a 24 h incubation period, 10 *μ*L of MTT solution (5 mg/mL in PBS) was added, followed by 3 h incubation at 37°C. Finally, media were aspirated and dimethyl sulfoxide (DMSO, 100 *μ*L) was added to solubilize the formazan salt formed. The optical density was read at 570 nm.

### 2.12. Evaluation of Intestinal Transit

Fasted female Swiss mice were orally treated with vehicle (Veh: water, 1 mL/kg), atropine (Atro: 3 mg/kg, s.c.), or HEAS (100 mg/kg) 30 min prior to administration of 0.5 mL of a semisolid marker solution (0.05% phenol red plus 1.5% carboxymethyl cellulose). After 20 min, the mice were euthanized and small intestine was dissected out from the pylorus to the ileocecal junction to the measurement of the intestinal transit. The total length of the small intestine and the distance covered by phenol red solution were then measured. Intestinal transit was expressed in percentage as calculated from IT = *X*/*Y* × 100, where *X* is distance traveled by phenol red and *Y* is total length of the small intestine.

### 2.13. Preliminary Toxicological Analysis

Mice were randomly divided into two groups (*n* = 10) and orally treated, once a day for 7 days, with vehicle (water, 10 mL/kg) or HEAS (100 mg/kg, p.o). The animals were observed daily to verify toxic signs of a general nature and the survivors were euthanized at the end of the treatment period. The spleen, liver, heart, lung, kidneys, and liver were excised, dried, and weighed precisely. For each organ, the relative weight was calculated as weight of organ × 100/body weight of mice on the day of the euthanasia.

### 2.14. Statistical Analysis

The data were expressed as means ± SEM. Differences between means were determined by one- or two-way analysis of variance (ANOVA) followed by Bonferroni's* post hoc* test, when applicable. The Mann-Whitney test was used in nonparametric data. The software GraphPad Prism 5® was used and *P* < 0.05 was considered to be significant in all experiments.

## 3. Results

### 3.1. Phytochemical Analysis

As described above, the phytochemical profile of the HEAS has been previously demonstrated by Barioni et al. [14]. In that occasion, using high performance liquid chromatography (HPLC) system, the two main compounds in HEAS were identified as luteolin and quercetin. Therefore, in this study, to standardize the HEAS, an estimation of the total phenol and flavonoid content was done.  Table 3 shows the total phenolic compounds and flavonoid contents in HEAS, which is possible to verify that 200 *μ*g of the extract represents 141.03 ± 1.43 *μ*g of tannic acid equivalents and 13.64 ± 0.74 *μ*g of quercetin equivalents.

### 3.2. HEAS Attenuated the Signs of DSS-Induced Acute Colitis

**Figure 2:**
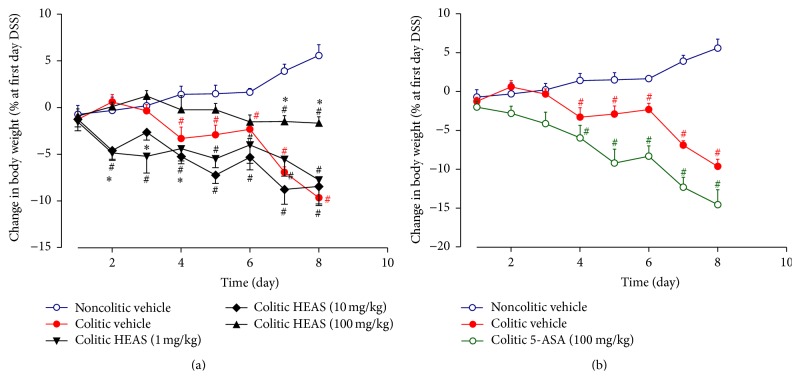
Effects of HEAS (1–100 mg/kg, (a)) or 5-ASA (100 mg/kg, (b)) on weight loss evolution. Data are expressed as mean ± SEM (*n* = 10). Statistical comparison was performed using two-way ANOVA followed by Bonferroni's test. ^#^ *P* < 0.01 versus noncolitic group. ^*∗*^ *P* < 0.01 versus control-colitic group.

**Figure 3:**
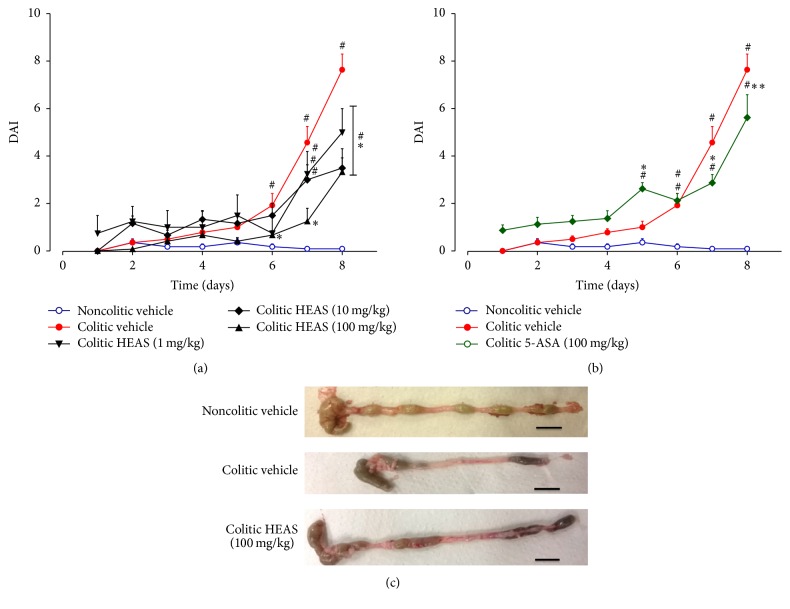
Effects of HEAS (1–100 mg/kg (a)) or 5-ASA (100 mg/kg (b)) on DAI values over the whole experimental period. (c) Macroscopic appearance of colon from mice treated with vehicle or HEAS (100 mg/kg). Data are expressed as mean ± SEM (*n* = 10). Statistical comparison was performed using two-way ANOVA followed by Bonferroni's test. ^#^ *P* < 0.01 versus noncolitic group. ^*∗∗*^ *P* < 0.01 and ^*∗*^ *P* < 0.01 and versus control-colitic group.

To evaluate the anti-inflammatory effects of HEAS, we used a mouse model of DSS-induced acute colitis, which mimics the acute phase of human UC. In the same way as Utrilla et al. [19], we analyzed various clinical signs of colitis and quantify such data using the DAI scoring system. As expected, on day 8, colitic mice treated with vehicle showed 6.3% loss of body weight (Figure 2(a)) and the DAI score was increased up to 8, corresponding to severe diarrhea and gross anal bleeding (Figure 3(a)). On the other hand, colitic mice treated with 1, 10, and 100 mg/kg of HEAS showed loss of body weight (Figure 2(a)) and DAI score significantly lower than colitic group treated with vehicle (Figure 3(a)). Unsurprisingly, the DAI score was also reduced in the colitic group treated with 5-ASA, the positive control drug of the experiment (Figure 3(b)). However, the treatment with 5-ASA did not inhibit the loss of body weight elicited by DSS intake (Figure 2(b)).  Figure 3(c) illustrates macroscopic observations of colon after the treatment with vehicle or HEAS (100 mg/kg). In addition, an improvement of the macroscopic appearance of the colon from colitic mice treated with HEAS (10 mg/kg) also was observed (data not shown).

**Figure 4:**
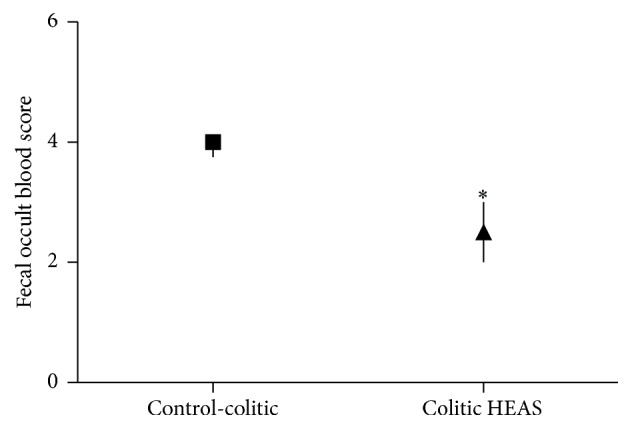
Effects of HEAS (100 mg/kg) on fecal occult blood score in DSS-induced colitic mice. Data are expressed as median ± interquartile range of triplicated experiments. Statistical comparison was performed using Mann-Whitney test. ^*∗*^ *P* < 0.05 versus control-colitic group.

Classically in this model, the shortening of the colon length indicates the extent of colon damage in experimental animals. Indeed, as observed in Table 4, noncolitic group showed an average colon length of 102.3 ± 3.7, whereas colitic group treated with vehicle exhibited a reduction in colon length to 62.5 ± 4.0 mm. Additionally, the weight of the colon also was reduced by 46% in colitic group treated with vehicle when compared with noncolitic group (1.73 ± 0.07 g/100 g, Table 4). Confirming the beneficial effects of the extract on DSS-induced colitis, HEAS (100 mg/kg) significantly improved the colon length and the weight of the empty colon to 84.0 ± 3.1 mm and 1.49 ± 0.14 g/100 g, respectively (Table 4), and, parallely, was also able to reduce the presence of occult blood in feces (Figure 4). In addition, HEAS, at dose of 10 mg/kg, also diminished the colon shortening evoked by DSS exposure in 19%.

According to Zhang et al. [29], the spleen of mice from colitic group treated with vehicle was markedly swollen, reaching an increase in organ weight of 56% when compared to noncolitic group (0.41 ± 0.02 g/100 g), and this alteration was not detected in colitic mice treated with HEAS at doses of 1 or 100 mg/kg (Table 4). In contrast, no difference in liver weight was perceived between the experimental groups (Table 4).

### 3.3. HEAS Decreased Histopathological Changes in the Colon Tissue of Mice with DSS-Induced Colitis

**Figure 5:**
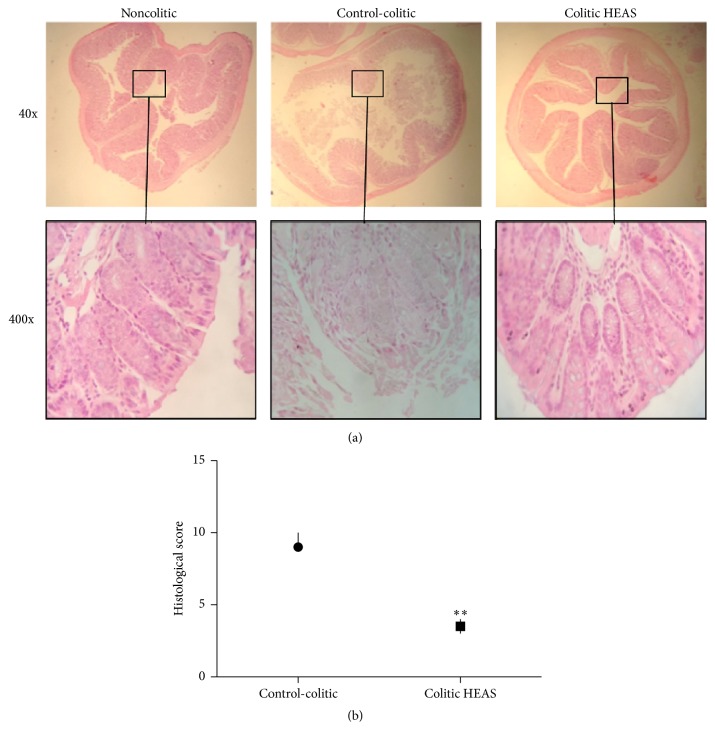
Effects of HEAS (100 mg/kg) on histological changes in colon tissue of DSS-induced colitic mice. (a) Representative images of all groups. (b) Values of histological changes. Data are expressed as median ± interquartile range (*n* = 10). Statistical comparison was performed using Mann-Whitney test. ^*∗∗*^ *P* < 0.01 versus control-colitic group.

The architecture of the colon from mice of different experimental group was accessed using HE staining. As expected, in noncolitic mice the structures of colon wall, submucosa, and crypts were normal (Figure 5(a)). In contrast, the colon of colitic group treated with vehicle exhibited pathological changes with loss of epithelial barrier, decrease in the number of crypts, and Goblet cells depletion. These pathological changes in colon were ameliorated by HEAS (10 and 100 mg/kg) treatment (Figure 5(a)). The microscopic appearance of the colon from colitic mice treated with HEAS (100 mg/kg) is showed in Figure 5(a). Histological changes were evaluated in samples from the noncolitic group and colitic group treated with vehicle or treated with the extract at a dose of 100 mg/kg, using a standard scoring system. As showed in Figure 5(b), the histopathological scores of HEAS-treated colitic group were significantly lower in relation to colitic group treated with vehicle (Figure 5(b)).

### 3.4. HEAS Prevents Mucin Depletion in the Colon Tissue of Mice with DSS-Induced Colitis

**Figure 6:**
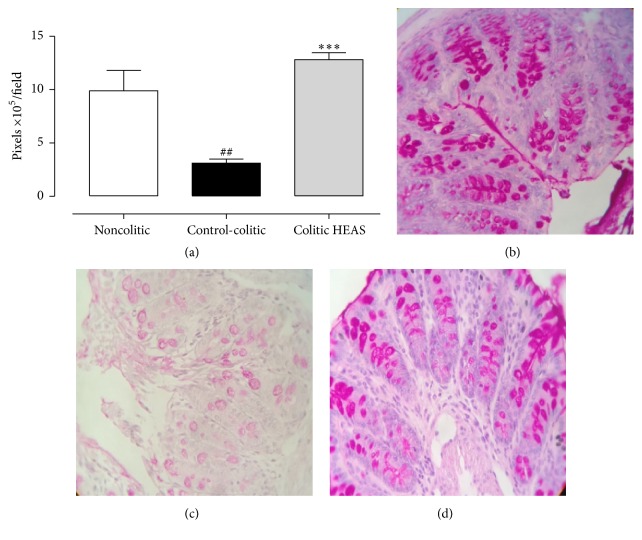
Effects of HEAS (100 mg/kg) on colonic staining for mucin-like glycoproteins. (a) Mucin staining is expressed as mean ± SEM (*n* = 10); statistical comparison was performed using one-way ANOVA followed by Bonferroni's test; ^##^ *P* < 0.01 versus noncolitic group and ^*∗∗∗*^ *P* < 0.001 versus control-colitic group. Representative image of noncolitic group in (b), control-colitic group in (c), and colitic HEAS group in (d). ((b)–(d)) Magnification*  *=* * 400x.

PAS histochemical staining is a classical technique used to detect the presence of glycoproteins, such as mucins, which are presented within cytoplasmic granules of the Goblet cells and protect the intestinal mucosa. As observed in Figure 6(a), the PAS staining in the colon from colitic mice treated with vehicle was decreased in 69% when compared to naive group (Veh: 9.88 ± 1.92 × 10^5^ pixels/field). Interestingly, HEAS (100 mg/kg) increased the PAS staining by 313% when compared to colitic vehicle group (Veh: 3.10 ± 0.39 × 10^5^ pixels/field). Microscopic observations of the PAS staining in colonic mucosa from naive, vehicle colitic group, and HEAS (100 mg/kg) colitic group are represented in Figures 6(b), 6(c), and 6(d), respectively. Besides, the administration of HEAS at 10 mg/kg also increased significantly the PAS staining at colitic mucosa; on the other hand, the HEAS at 1 mg/kg was not be able to provoke this increase (data not showed).

### 3.5. HEAS Improves Oxidative Stress in Colon of Mice with DSS-Induced Colitis

The oxidative stress contributes to intestinal inflammation in IBD patients and in animal models of experimentally induced colitis [30, 31]. In fact, the availability of the antioxidant GSH and the SOD activity were decreased in colon tissue of colitic mice treated with vehicle in 43% and 89%, respectively, when compared to noncolitic group (797.50 ± 111.10 *μ*g of GSH/mg of tissue and 1.36 ± 0.22 U SOD/mg of protein) (Table 5). Additionally, LOOH levels were increased in 97% in colitic mice treated with vehicle, when compared to noncolitic group (2.03 ± 0.58 mmol of LOOH/mg of tissue, Table 5). The treatment with HEAS (10 or 100 mg/kg) or 5-ASA (100 mg/kg) did not change GSH or LOOH levels in colon tissue but fully restored the SOD activity to basal levels, as demonstrated in Table 5.

### 3.6. HEAS Features Scavenger Effect of Free Radicals

**Figure 7:**
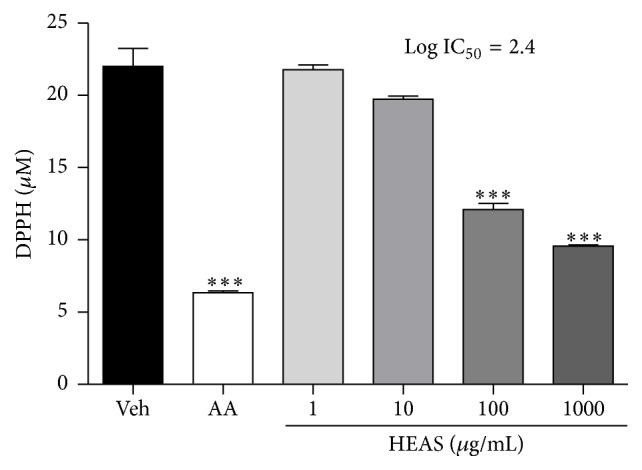
*In vitro *ability of HEAS (1–1000 *μ*g/mL) to scavenge the free radical DPPH. The results are expressed as mean ± SEM of triplicated experiments. Statistical comparison was performed using one-way ANOVA followed by Dunnett's test. ^*∗∗∗*^ *P* < 0.001 versus vehicle group (Veh). AA: ascorbic acid (50 *μ*g/mL).

Corroborating the* in vivo* antioxidant capacity of HEAS, the* in vitro* DPPH assay showed that HEAS concentration dependently scavenged DPPH radicals, with a log IC_50_ = 2.4 (Figure 7). As expected, ascorbic acid (the standard control) reduced DPPH levels by 70.74% when compared with vehicle (22.01 ± 1.24 *μ*M; Figure 7).

### 3.7. HEAS Decreases the MPO Activity in the Colon Tissue of Mice with DSS-Induced Colitis

MPO is an enzyme present in neutrophils and in smaller quantities in monocytes and macrophages. Classically, the MPO activity is used as a marker of acute inflammation by its relation to the degree in neutrophil infiltration. The MPO activity in colonic tissues of colitic mice treated with vehicle was increased up to 268%, when compared to noncolitic group (1.15 ± 0.50 mD.O/mg of protein) (Table 5). On the other hand, the treatment with HEAS (10 mg/kg) reduced the MPO activity in 30%, whereas the treatment with the extract, at the highest dose (100 mg/kg), was able to restore the levels of this parameter to basal values (Table 5). Nevertheless, the treatment with 5-ASA (100 mg/kg) did not significantly reduce the MPO activity, compared to vehicle colitic group.

### 3.8. HEAS Decreases TNF-*α* and IL-6 Levels and Increases IL-10 Levels in the Colon of Mice with DSS-Induced Colitis

**Figure 8:**
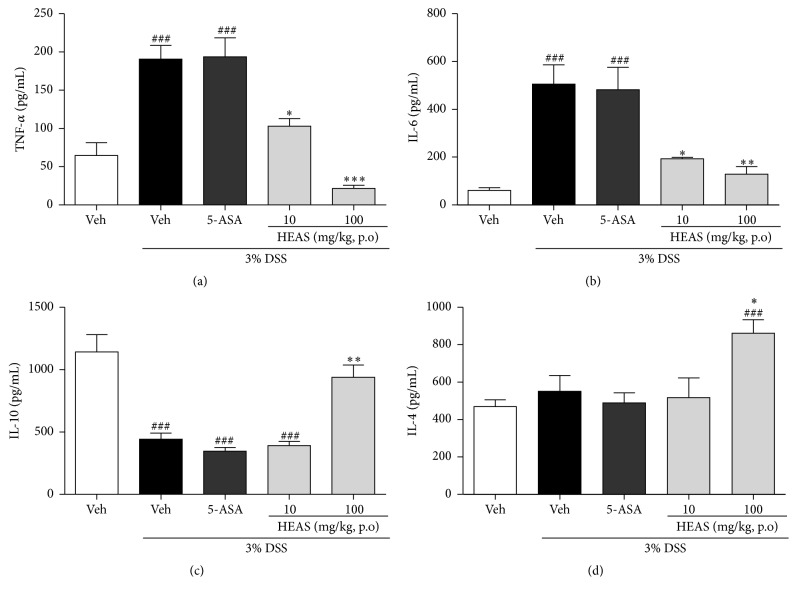
Effects of HEAS (100 mg/mL) on TNF-*α* (a), IL-6 (b), IL-10 (c), and IL-4 (d) levels in colon tissue of DSS-induced colitic mice. The results are expressed as mean ± SEM (*n* = 10). Statistical comparison was performed using one-way ANOVA followed by Bonferroni's test. ^###^ *P* < 0.001 versus noncolitic group. ^*∗*^ *P* < 0.05, ^*∗∗*^ *P* < 0.01, and ^*∗∗∗*^ *P* < 0.001 versus control-colitic group.

DSS administration significantly increased the levels of TNF-*α* (Figure 8(a)) and IL-6 (Figure 8(b)) in colon tissue by 193 and 726% in relation to the noncolitic group (64.85 ± 16.55 pg/mL and 61.10 ± 11.55 pg/mL, resp.). In contrast, DSS treatment decreased the content of IL-10 (Figure 8(c)) in colon in 61%, when compared to noncolitic group (1144.01 ± 138.10 pg/mL). The treatment with HEAS, at doses of 10 and 100 mg/kg, reduced the levels of TNF (Figure 8(a)) in 46 and 62%, respectively, compared to colitic vehicle group. Similarly, the levels of IL-6 (Figure 8(b)) were decreased in 62 and 75% in colitic mice treated with HEAS, at doses of 10 and 100 mg/kg, respectively, related to colitic vehicle group. In addition, the daily administration of HEAS (100 mg/kg) increased the levels of IL-4 (Figure 8(d)) and IL-10 (Figure 8(c)) in 40 and 112%, respectively, when compared to vehicle group exposed to DSS.

### 3.9. HEAS Reduces the LPS-Induce NO Production in Isolated Rat Peritoneal Macrophage

**Figure 9:**
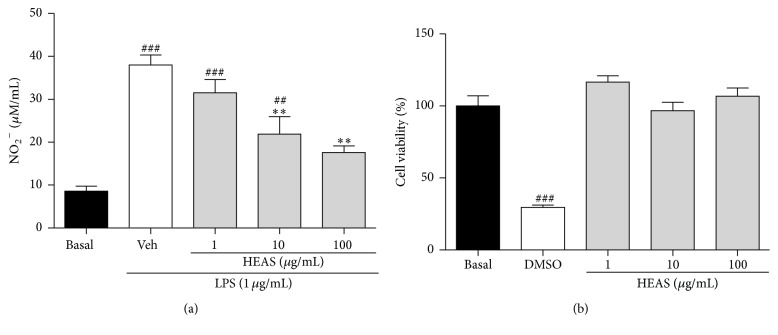
Effects of HEAS (1–100 *μ*g/mL) on the LPS induced NO production in isolated rat peritoneal macrophage (a) and on the cellular viability (b). The results are expressed as mean ± SEM of triplicated experiments. Statistical comparison was performed using one-way ANOVA followed by Bonferroni's test. ^###^ *P* < 0.001 and ^##^ *P* < 0.01 versus basal group. ^*∗∗*^ *P* < 0.01 versus vehicle group (Veh).

To investigate whether HEAS has an anti-inflammatory activity, NO production was determined in the presence of the extract at 1, 10, or 100 *μ*g/mL in LPS-induced peritoneal macrophages. When LPS were incubated to peritoneal macrophages, the nitrite (a surrogate of NO production) generation dramatically increased from the basal level of 8.54 to 37.97 *μ*M after 24 h incubation (Figure 9(a)). LPS-induced nitrite generation was significantly and concentration dependently attenuated by HEAS up to 50% at 100 *μ*g/mL (Figure 9(a)). In addition, this inhibitory effect of HEAS was not triggered by nonspecific cytotoxicity, because the extract had no effect on cell viability as determined by MTT assay at concentrations from 1 to 100 *μ*g/mL (Figure 9(b)).

### 3.10. HEAS Unchanged the Intestinal Transit in Mice

**Figure 10:**
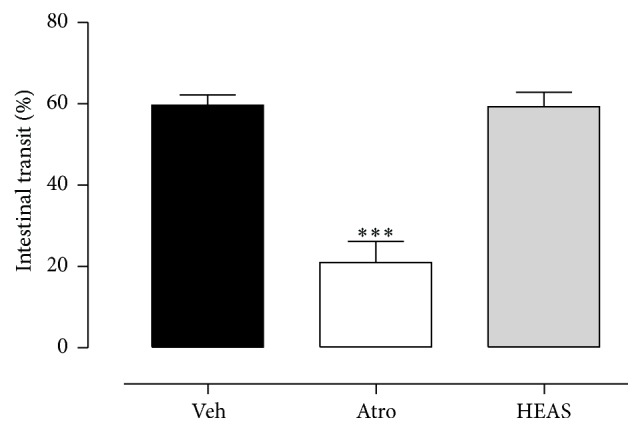
Effects of HEAS (100 mg/mL) on intestinal transit rate of mice. The results are expressed as mean ± SEM (*n* = 8). Statistical comparison was performed using one-way ANOVA followed by Bonferroni's test. ^*∗∗∗*^ *P* < 0.001 versus vehicle group (Veh).

The rate of intestinal transit of semisolid phenol red after 15 min in control mice was 62.33 ± 3.56% and the oral treatment with HEAS (100 mg/kg) did not modify this parameter. Expectedly, atropine (the positive control) reduced this rate to 21.02 ± 5.20% (Figure 10).

### 3.11. HEAS Did Not Promote Any Signs of Toxicity in Mice

**Figure 11:**
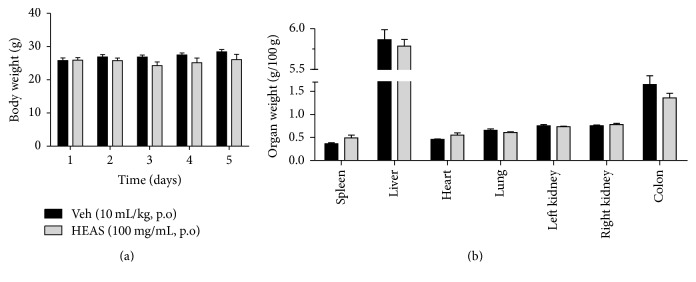
Effects of HEAS (100 mg/mL) on the body weight (a) and on the relative organs weight (b) of mice. The results are expressed as mean ± SEM (*n* = 10). Statistical comparison was performed using two-way ANOVA followed by Bonferroni's test.

The oral administration of HEAS (100 mg/kg) did not promote any signs of toxicity in mice and in accordance did not change the body weight (Figure 11(a)) or the organ weight (Figure 11(b)) of mice, comparable to the vehicle treated group.

## 4. Discussion

In our previous studies, the hydroalcoholic extract of inflorescences of* A. satureioides *(HEAS) displayed gastroprotective effect against different harmful agents, without signs of toxicity after its administration at a dose of 2000 mg/kg [15, 32]. In agreement, the ability of this extract to inhibit neutrophil functions related to the innate response has also been described [14]. In the current trial, the intestinal anti-inflammatory effect of HEAS was confirmed in a mouse model of DSS-induced colitis. DSS intake was intended to induce clinical features in mice that are similar to mild colitis, including impaired barrier function, intestinal epithelial cell inflammation, and oxidative stress [33]. We found that HEAS improves DSS-induced experimental colitis by reduction of inflammatory and oxidative damage in colon, in a dose dependent-manner, mainly through the attenuation of inflammatory cytokines production and the improvement of intestinal mucin barrier.

**Figure 12:**
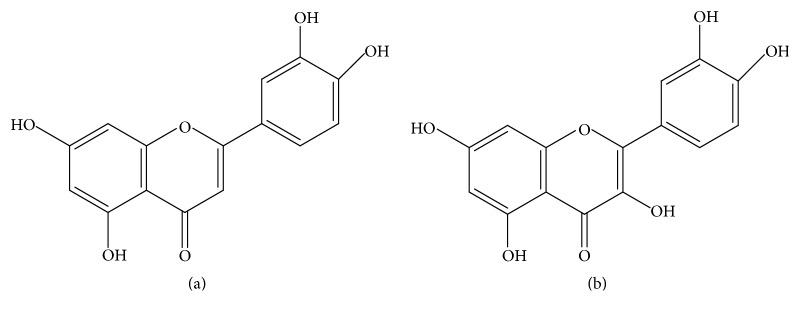
(a) Luteolin structure; (b) quercetin structure.

The inflorescences of* A. satureioides *have traditionally been used in South American countries to prevent diseases and reduce inflammation, including gastrointestinal disorders [9]. Numerous studies have reported the bioactive properties of* A. satureioides*, including antioxidant [34], antimicrobial [35], anti-inflammatory [11, 13, 14], gastroprotective [15, 32], insecticidal [36], antiherpetic [37], and trypanocidal [38] activities. The functional properties of natural agents are associated with their phytochemistry composition and the presence of the main compounds luteolin (yield 2.28%) (Figure 12(a)) and quercetin (yield 2.53%) (Figure 12(b)) was previously confirmed by phytochemicals analysis [14, 32]; besides, GC-MS analysis showed that HEAS also contains steroids and fatty acids [14]. Indeed, the intestinal anti-inflammatory activity of luteolin [38] and quercetin [39] was confirmed by* in vivo* and* in vitro* experiments. In this study, the total phenol and flavonoids in HEAS were measured revealing that the amount of these compounds represents 70.51% and 6.82% of HEAS, respectively. The flavonoids are pointed out as having beneficial effects to treat IBD [40]. The luteolin plus quercetin content in HEAS was previously reported by Barioni et al. [14] equal to 4.81%, which is consistent with the total flavonoid content reported in the results of this study. Thus, these findings confirmed and extended the phytochemical profile of the extract, contributing quantitative data to the validation of herbal product from inflorescences of* A. satureioides *to treat IBD based on flavonoid content.

Based on these observations, we hypothesized that HEAS presents anti-inflammatory effect against experimental colitis and may represent a source of a new herbal product based on traditional knowledge for the treatment of IBD. As expected, the results of DAI and the findings of histological examination confirmed this purpose. Furthermore, the results showed in this study pointed out beneficial effects of the extract in DAI values even at the lowest dose.

In the colitis induced by DSS intake, the positive control agent used was 5-ASA, which is anti-inflammatory aminosalicylate used in the management of IBD in humans [41]. Unsurprisingly, the treatment with 5-ASA improved DAI scores and these findings improve the accuracy and the validity of the experimental results showed in this study.

The spleen is a peripheral immune organ with a wide variety of immune cells; it is known that a number of infections and diseases can contribute to an enlarged spleen tissue [42]. Regarding colitis, Zhang et al. [29] have reported that the spleen is markedly swollen in DSS colitic animals. Thus, increased spleen weight generally correlates with the extent of inflammation and anemia in this model [18]. Our study also detected an augmented spleen weight in DSS-induced colitis in mice, which was prevented by HEAS treatment. In view of the above observation, we can infer that HEAS has benefic effects on the immune response to the whole body.

A continuously secreted mucus layer formed by high molecular weight oligomeric mucin glycoproteins, which exert important role in mucosal defense [43], lines the gastrointestinal tract. Nowadays there are many reasons to believe that the damage in mucosal barrier is critically important to the pathogenesis of IBD [44]. Herein, the colonic mucin levels were quantified based upon their PAS reactivity and this measurement confirmed the depletion in Goblet cells at DSS-injured colon. Consistent with the improvement evoked by HEAS in inflamed colon, the depletion in mucin content was minimized in the group treated with the extract (100 mg/kg). It is known that injuries in mucosal barrier function are linked to modifications in macromolecules permeability by colonic epithelium, increasing bacterial invasion and/or translocation [45]. These events can trigger the release of inflammatory and oxidative mediators, leading to the damage in colon; in contrast, the maintenance of the integrity of the mucin barrier promoted by protective effects of HEAS could result in a primary event in beneficial mode of action of this extract.

Oxidative stress plays an important role in the pathogenesis of inflammatory bowel diseases and occurs when the production of reactive oxygen species (ROS) exceeds tissue antioxidant resources [46]. In inflamed colonic tissue, ROS are produced by activated neutrophils and macrophage and its production can be inferred by measuring the levels of LOOH [47]. In fact, colitic mice treated with vehicle exhibited increased LOOH content and this change was accompanied by reduction in GSH levels, a nonenzymatic antioxidant defense. In addition, similarly to Zhao et al. [48], the SOD activity was reduced in colon tissue from colitic mice treated with vehicle. SOD is metal ion cofactor-requiring enzymes that catalyze dismutation of superoxide anion (O_2_^•−^) into molecular oxygen (O_2_) and hydrogen peroxide (H_2_O_2_) [47]. Collectively, these findings characterized intense oxidative damage at colonic sites caused by DSS intake. Considering the effects of HEAS, the measurement of oxidative parameters was performed in samples from colitic mice treated with HEAS at doses of 10 and 100 mg/kg, but not 1 mg/kg. On the other hand, treatment with HEAS (10 and 100 mg/kg) promoted beneficial effects in SOD activity and in LOOH content in samples from colitic mice. However, HEAS treatment did not elicit any effect on GSH depleted levels. Nevertheless, the* in vitro* DPPH radical scavenging profile presented by HEAS confirmed its scavenging effect and is likely, but not yet proven, that this ability compensates the depleted GSH. Moreover, the reduction in LOOH in colonic tissue is a remarkable evidence of the improvement of intracellular redox status during the anti-inflammatory action promoted by HEAS.

In addition, HEAS (10 and 100 mg/kg) also reduced neutrophils infiltration in colon tissues, which was evidenced by reduction in MPO activity. MPO is an index of neutrophil recruitment in the DSS-induced colitis model and in consequence reflects the inflammatory events in parallel with cytokine concentrations. Indeed, the colonic MPO activity was significantly increased in the colitic vehicle treated group compared with the noncolitic group, which was decreased by HEAS treatment. These results extend and reinforce those described by Barioni et al. [14], at which the administration of* A. satureioides* extract reduced* in vivo* LPS neutrophil influx to the exudates at air pouch model and the number of rolling and adhered leukocytes in LPS-stimulated mesentery. In addition, the authors have confirmed that the* in vivo* treatment with* A. satureioides* extract modifies the adhesive properties of neutrophils to the endothelium, which then impair their migration into inflamed tissue. Accordingly, HEAS also reduced neutrophil infiltration into injured colon tissue and we can suggest that these effects described by Barioni et al. [14] are also involved in the intestinal anti-inflammatory actions elicited by HEAS.

The complete etiology of IBD is unknown, but there is a consensus that along with the increased recruitment of leukocytes to the site of inflammation, also there is an imbalanced production of proinflammatory mediators [49]. Particularly, the increase in inflammatory cytokines secretion in intestinal mucosal is unselectively increased in both ulcerative colitis and Crohn's disease [50]. In accordance to that previously demonstrated by Sreedhar et al. [51], DSS intake induced an increase in colonic TNF-*α* and IL-6 levels, which was associated with colon damage. However, in our results the levels of Th2 cytokine IL-4 were not significantly increased (*P* = 0.1582) in colitic mice. In agreement, no difference was found in IL-4 release in experimental colitis by Dieleman et al. [52] and by Barros et al. [53] in the acute phase of ulcerative colitis. Interestingly, the treatment with HEAS, at the major dose tested, promoted an increase in IL-4 levels. Furthermore, reinforcing the beneficial effects of HEAS, the treatment with the extract, at doses of 10 and 100 mg/kg, decreased TNF-*α* and IL-6 content in colon tissue at basal levels. Moreover, it is described that IL-10 acts as a key mediator for maintaining gut homeostasis and that sequence variants in the IL-10 locus contribute to UC. In accordance, HEAS (100 mg/kg) treatment also normalized the secretion of IL-10. IL-10 is an immunoregulatory cytokine involved in the innate and cell-mediated response [54]. It promotes the downregulation of colon inflammation by the inhibition of both antigen presentation and release of proinflammatory cytokines [55] and is directly related to regulatory cells activity [56].

*In vitro* analyses related to the anti-inflammatory effects of HEAS also were performed. Macrophage activation induced by LPS increased the production of proinflammatory cytokines and inflammatory mediators, including NO. Indeed this effect was assessed in our study and HEAS was able to inhibit NO production in LPS-stimulated peritoneal macrophages. Cytotoxicity assay performed with HEAS incubation indicated that the extract even at dose of 100 *μ*g/mL did not affect the viability of peritoneal macrophages. Therefore, suppressive effects in NO production are not due to its cytotoxic effects. On the basis of the* in vitro* study, the inhibition of the NO production by HEAS can be a result from the suppression of the enzymatic activities and/or expression levels of inducible nitric oxide synthase isoform. Furthermore, DSS-induced colitis might be improved by suppressing macrophage activation through HEAS treatment. This hypothesis is especially strengthened by the elevated IL-4 levels found in colon samples from colitic mice treated with HEAS (100 mg/kg).

Regarding the experiment evaluating intestinal motility, HEAS administration at the same dose that reduces inflammation associated with DSS-induced colitis did not produce any change in intestinal transit rate. This result is important since it indicates that HEAS effects, especially in diarrhea parameter, are not associated with a reduction in intestinal motility. Taking into account that the reduction in the body and in the relative organs weight is a simple and sensitive marker of toxicity of substances, the data obtained here provide preliminary information about the safety of the HEAS.

## 5. Conclusion

The hydroalcoholic extract of inflorescences of* A. satureioides *displayed intestinal anti-inflammatory activity in the DSS-induced colitis model by the maintenance of intestinal mucin barrier, reducing the neutrophil migration and macrophage activation, and consequently the oxidative damage, in parallel to the adjustment in the levels of pro- and anti-inflammatory cytokines. Together, these results suggest* A. satureioides* as a promising source of herbal medicine that could be used in treatment of the inflammatory bowel disease. Besides, this work corroborates the popular use of* A. satureioides* in inflammatory disorders.

## Figures and Tables

**Table 1:** DAI Score.

Score	Weight loss	Stool consistency	Rectal bleeding
0	None	Normal	Normal
1	1–5%	—	—
2	5–10%	Loose stools	—
3	10–20%	—	—
4	>20%	Diarrhea	Gross bleeding

DAI value is composed by the sum of the scores of weight loss, stool consistency and rectal bleeding. Maximum score: 12.

**Table 2:** Variables used in the histological score.

Findigns	Score	Criteria
Mucosal epithelium	0	No mucosa inflammation
	1	Loss of <5% of the epithelial surface
	2	Loss of 5–10% of the epithelial surface
	3	Loss of >10% of the epithelial surface

Integrity of crypts	0	Intact crypts
	1	Loss of <10% of crypts
	2	Loss of 10–20% of crypts
	3	Loss of >20% of crypts

Cell infiltrate and edema	0	None
	1	Mild
	2	Moderate
	3	Severe

Goblet cells depletion	0	Absent
	1	Present

Histological score value is composed by the sum of the scores of all variables. Maximum score: 10.

**Table 3:** Total phenolic content and flavonoid amount of HEAS.

Extract (*μ*g)	T.A.E. ± S.E.M.	Q.E. ± S.E.M.
200	141.03 ± 1.43	13.64 ± 0.74
150	121.51 ± 1.82	10.51 ± 1.07
100	87.14 ± 0.95	8.45 ± 0.09
50	46.46 ± 1.24	n.d

The results are expressed as means ± S.E.M. (*n* = 3) of tannic acid equivalents (T.A.E.) in *μ*g or quercetin equivalents in *μ*g (Q.E.). n.d = not detected.

**Table 4:** Effects of HEAS on lenght and weight of colon, and on weight of spleen and liver of DSS-induced colitic mice.

	Treatment	Colon length (mm)	Colon weight (g/100 g)	Spleen weight (g/100 g)	Liver weight (g/100 g)
Non-colitic	Vehicle	102.3 ± 3.7	1.73 ± 0.07	0.41 ± 0.02	4.32 ± 0.29
Colitic	Vehicle	62.5 ± 4.0^a^	0.92 ± 0.11^a^	0.62 ± 0.06^a^	4.48 ± 0.14
Colitic	5-ASA (100 mg/kg)	84.0 ± 3.9^b^	1.15 ± 0.06^c^	0.41 ± 0.03^b^	4.56 ± 0.16
Colitic	HEAS (1 mg/kg)	72.5 ± 5.1	1.10 ± 0.04	0.48 ± 0.04^b^	4.53 ± 0.19
Colitic	HEAS (10 mg/kg)	81.8 ± 3.7^b^	0.95 ± 0.01	0.53 ± 0.04	4.47 ± 0.25
Colitic	HEAS (100 mg/kg)	84.0 ± 3.1^b^	1.49 ± 0.14^b^	0.49 ± 0.01^b^	4.70 ± 0.38

^a^ *P* < 0.001 versus non-colitic group; ^b^ *P* < 0.001 versus colitic group; ^c^ *P* < 0.05 versus colitic group.

**Table 5:** Effects of HEAS on MPO and SOD activity and on GSH and LOOH levels in colon tissue of DSS-induced colitic mice.

	Treatment	Dose (mg/kg, p.o)	MPO (mD.O/mg of protein)	SOD (U/mg of tissue)	GSH (*µ*g/mg of tissue)	LOOH (mmol/mg of tissue)
Non-colitic	Vehicle	—	1.15 ± 0.50	1.36 ± 0.22	797.50 ± 111.10	2.03 ± 0.58
Colitic	Vehicle	—	4.24 ± 0.71^a^	0.15 ± 0.03^a^	454.70 ± 78.24^a^	3.99 ± 0.60^a^
Colitic	5-ASA	100	3.80 ± 0.24^a^	1.10 ± 0.10^b^	424.78 ± 59.28^a^	4.02 ± 0.29^a^
Colitic	HEAS	10	2.96 ± 0.11^a,b^	1.35 ± 0.04^b^	444.12 ± 18.42^a^	3.52 ± 0.42^a^
Colitic	HEAS	100	1.26 ± 0.36^b^	1.36 ± 0.64^b^	482.70 ± 62.52^a^	4.14 ± 0.34^a^

^a^ *P* < 0.001 versus non-colitic vehicle group, ^b^ *P* < 0.01 versus colitic vehicle group.
